# Primary Diffuse Large B Cell Lymphoma Mimicking Hyperplastic Reactive Lesion (Lymphoma of the Oral Cavity)

**DOI:** 10.1155/2018/2981689

**Published:** 2018-02-07

**Authors:** Liziane Cattelan Donaduzzi, Angélica Reinheimer, Maria Augusta Ramires da Silva, Lucia de Noronha, Aline Cristina Batista Rodrigues Johann, Ademir Franco, Soraya de Azambuja Berti Couto, Paulo Henrique Couto Souza

**Affiliations:** ^1^School of Life Sciences, Department of Dentistry, Pontifícia Universidade Católica do Paraná, Curitiba, PR, Brazil; ^2^School of Medicine, Pontifícia Universidade Católica do Paraná, Curitiba, PR, Brazil

## Abstract

**Objective:**

To report a case of a challenging oral diagnosis involving a primary diffuse large B cell lymphoma of the oral cavity mimicking a hyperplastic reactive lesion.

**Case Report:**

A 72-year-old male patient was referred to the Department of Stomatology presenting a proliferative nodular lesion in the anterior region of the mandible involving the anterior teeth. The clinical examination revealed anterior teeth affected by periodontal disease, suggesting the nodular cession hyperplastic reaction. Incisional biopsy was performed under local anesthesia. The histopathological examination revealed a diffuse proliferation of atypical large lymphoid cells. The tumor cells showed immunopositivity for CD20 and Ki67 (100%) and negativity for CD3, CD30, and CD15. The diagnosis of diffuse large B cell lymphoma was established. The patient underwent chemotherapy and progressed to death after nine months.

**Conclusion:**

Lymphomas of the oral cavity are rare and may have nonspecific clinical features, mimicking inflammatory and reactive lesions. Therefore, a detailed clinical evaluation associated with histopathological and immunohistochemical analysis should be performed to enable early and accurate diagnoses in suspected oral lesions.

## 1. Introduction

Lymphomas are unusual malignant tumors that affect the lymph reticular system characterizing a proliferation of lymphoid clone somewhat differentiated. These tumors are morphologically divided into two groups: Hodgkin's lymphoma (HL) and non-Hodgkin's lymphoma (NHL) [1]. The first involves predominantly the lymph glands. Specifically, only 1% affects extra nodal sites, contrasting with 23–30% of the NHL [2]. Head and neck comprehend the second region most affected by NHL after the gastrointestinal tract. The involvement of the oral cavity is rare and accounts for only 3.5% of the cases reported. Although rare, they are the second mostly common malignancy in the mouth, constituting 2.2% of head and neck tumors [3].

The various types of lymphoid neoplasms originated from B and T cell populations are reported. These neoplasms have different clinical, morphological, and molecular characteristics, making their classification complex. Currently, the most used classification is proposed by the World Health Organization (WHO) [1], which suggests more than 30 subtypes of NHL categorized according to their growth rate (indolent, aggressive, and highly aggressive). The diffuse large B cell lymphoma (DLBCL) is the most prevalent in Western countries, accounting for approximately 30% of newly diagnosed cases [4]. The DLBCL affects both hard and soft tissues with a preference for tonsils, palate, and parotid glands [5, 6]. The involvement of gingival tissue is rare because the lymphoid tissue is not normally found in this region [7]. Generally, DLBCL lesions have different clinical presentations, including local edema, exophytic proliferation, erythematous aspect, and ulceration. Radiographically, it may reveal bone loss [8]. Despite that, intraoral lymphomas may present nonspecific clinical characteristics, mimicking inflammatory and reactive lesions such as periodontal disease and pyogenic granuloma [9]. In this context, the DLBCL should be considered as differential diagnosis of proliferative lesions in the oral cavity [7].

Rapid growth of DLBCL lesions may be associated with the Acquired Immune Deficiency Syndrome (AIDS). Specifically, the immunosuppressant condition generated by the Human Immunodeficiency Virus (HIV) may trigger an abnormal proliferation of lymphocytes [7]. Therefore, attention should be given to apparently benign lesions in the oral cavity, especially in HIV positive patients [10].

The present study reports a case of DLBCL mimicking a reactive periodontal disease.

## 2. Case Presentation

**Figure 1:**
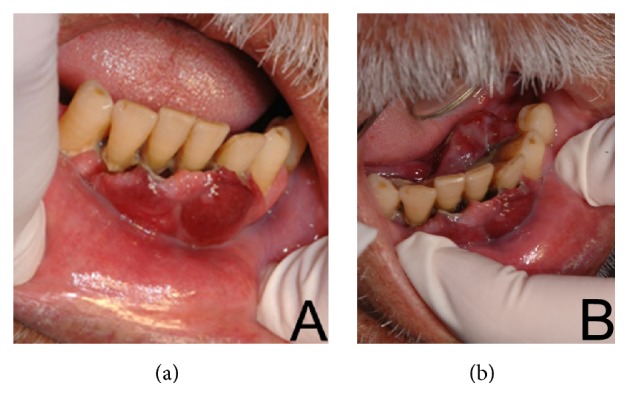
Nodular lobed and sessile lesion, with flabby consistency and dark red color covering the mandibular teeth, affected by periodontal disease (a), and extending to the floor of the mouth (b).

A 72-year-old male patient was referred to the Stomatology Department complaining of an increased gingival volume in the region of the mandibular anterior teeth. According to the patient, the lesion grew within one month. The patient also reported being alcoholic and a former smoker. His medical history included hypertension and diabetes mellitus type 2 and the patient reported a basal cell carcinoma detected five years ago. No additional information about infectious disease as C hepatitis or AIDS was reported. The extraoral examination revealed no lymphadenopathy. The intraoral examination revealed poor oral hygiene, general periodontal disease, and a sessile nodular lesion located on the buccal and lingual gingiva of the mandibular incisors extending to the floor of the mouth. The patient had lost all maxillary teeth in the past because of periodontal disease. The lesion presented a lobulated surface, flabby consistency, and dark red color, measuring 2 cm in diameter (Figure 1). The teeth involved had biofilm accumulation, calculus, and mobility, hampering masticatory functions. A panoramic radiograph showed significant horizontal bone loss adjacent to the anterior teeth, extending to the mandibular left first premolar (#34).

**Figure 2:**
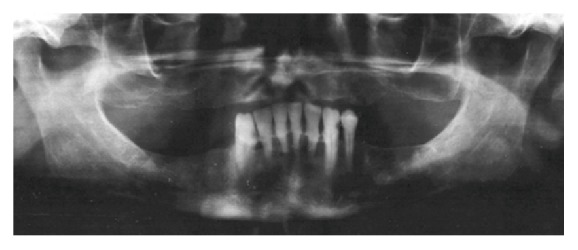
Panoramic radiograph showing horizontal bone loss in the region of mandibular anterior teeth, consistent with chronic periodontitis.

Accumulation of calculus was also observed, suggesting a condition of chronic periodontal disease (Figure 2). The hypothesis of a reactive hyperplastic lesion related to the severe gingival inflammation was considered. In this context, the potential diagnoses included pyogenic granuloma, focal fibrous hyperplasia, peripheral giant cell granuloma, and peripheral ossifying fibroma. Additionally, squamous cell carcinoma and lymphoma were also suspected in face of the rapid progression of the lesion.

**Figure 3:**
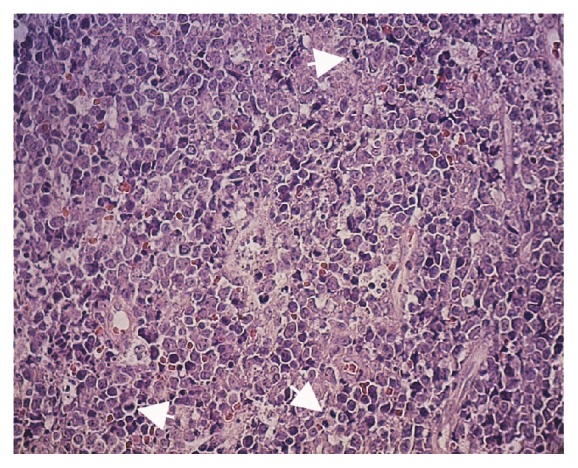
Histological aspect of the specimen revealing a diffuse proliferation of atypical large lymphoid cells, with predominance of centroblasts and high mitotic index (arrows) (HE ×100).

Considering these hypotheses, an incisional biopsy was performed under local anesthesia. The histopathological exam showed a diffuse proliferation of atypical large lymphoid cells, displaying numerous mitotic figures (Figure 3).

Predominantly, the neoplastic cells presented round nuclei, multilobate, or irregular folds, with looser chromatin with multiple nucleoli surrounded by membrane (centroblasts). Areas of necrosis were also observed. Immunohistochemical analysis revealed that tumor cells expressed immunopositivity for CD20 and Ki67 (100%) and negative expression for CD3, CD30, and CD15. The histology and immunohistochemistry were consistent with DLBCL. The patient was referred to an Oncological Hospital where he started chemotherapy but progressed to death in nine months.

## 3. Discussion

DLBCL is the most common subtype of non-Hodgkin's lymphomas affecting the jaw [11]. The presented case reported a histological manifestation of DLBCL compatible with previous descriptions [12]. Accordingly, the patient's age was also compatible with the affected age range reported in the literature, which includes patients aged above 50 years. The age related information became an important matter in the last decades considering the prevalence of DLBCL, because the survival rate of HIV positive patients gradually increases over the time. This data clearly suggests that HIV infection should be considered as a risk factor for lymphomas, highlighting the importance of investigating the patients' serology [13] in the routine of dentistry. In the present case a negative HIV serology was observed.

In most cases DLBCL has no specific etiology, being potentially related to genetic, environmental, and occupational influences [14]. Some of the most reported etiologies in the literature are (1) autoimmune diseases, such as Sjogren's syndrome and rheumatoid arthritis; (2) infectious agents, such as Epstein-Bar virus and bacteria* Helicobacter pylori*; and (3) occupational exposure to harmful chemical agents [15]. Defining the etiology of DLBCL may be difficult to establish, since the diagnosis of oral lymphomas has low rate of clinical suspicion because the lesions often mimic more common pathologies, such as periodontal disease [16]. In the present case, the potential diagnosis included giant cell granuloma, squamous carcinoma, and reactive injury caused by periodontal disease. Similarly to our case, another unusual case of gingival anaplastic large-cell lymphoma mimicking a benign hyperplastic lesion in a 57-year-old man as the first oral clinical manifestation of acquired immunodeficiency syndrome (AIDS) has been reported in the literature [10]. Other benignant oral lesions that might mimic potentially premalignant lesions, for instance, with sponge nevus, require careful clinical and histological examinations, avoiding misdiagnosis [17]. In face of the many potential diagnoses and considering the aggressive behavior of the lesion, the incisional biopsy was performed. In this context, the immunohistochemical analysis of the injury enabled appropriate decision on diagnosis and treatment plan. Specifically, the outcome for the expression of Ki-67 was 100% in the present case. The literature describes an expression range between 30 and 100%, characterizing an aggressive behavior when the immunopositivity rate reached above 80% [18]. It justifies the rapid growth of the lesion in a time interval of one month and explains the patient's progress to death within nine months.

Lastly, the recent literature has described the bioimpedance analysis as a noninvasive screening for potential precancerous lesions, as it could happen for any chronic diseases affecting the oral mucosa, representing a useful aid for the early detection of oral cancer in its preliminary stages. In the future, this alternative method may become a useful tool to carry out the diagnosis of oral lesions as described in the current paper [19].

## 4. Conclusion

The current case report shows how important is to manage an accurate clinical and histological examinations during the routine of dental practice, mainly by general clinicians. Usually, similar clinical cases would have been treated as advanced periodontal disease associated with proliferative gingival lesions. In these cases, general clinicians would proceed doing periodontal curettage followed by full surgical excision of the proliferative tissues without histological analysis, impairing the correct diagnosis of the lesion and the patient's treatment. Histological analysis of suspected oral lesions is always mandatory.

## References

[1] Swerdlow S., Campo E., Harris N. L., Jaffe E. S., Pileri S. A., Stein H. (2008). *WHO classification of tumour of haematopoietic and lymphoid tissues*.

[2] Shah G. H., Panwar S. K., Chaturvedi P. P., Kane S. N. (2011). Isolated primary extranodal lymphoma of the oral cavity: A series of 15 cases and review of literature from a tertiary care cancer centre in India. *Indian Journal of Medical and Paediatric Oncology*.

[3] Epstein J. B., Epstein J. D., Le N. D., Gorsky M. (2001). Characteristics of oral and paraoral malignant lymphoma: a population-based review of 361 cases. *Oral Surgery, Oral Medicine, Oral Pathology, Oral Radiology, and Endodontology*.

[4] Armitage J. (1997). A clinical evaluation of the international lymphoma study group classification of non-Hodgkin's lymphoma. *Blood*.

[5] Nogai H., Dörken B., Lenz G. (2011). Pathogenesis of non-Hodgkin's lymphoma. *Journal of Clinical Oncology*.

[6] Souto G.-R., Pereira T.-D., Castro A.-F., Mesquita R.-A. (2013). Diffuse large B-cell lymphoma, not otherwise specified of the palate: A case report. *Journal of Clinical and Experimental Dentistry*.

[7] Basavaraj K. F., Karthikeyan R., Sarkar A., Muddaiah S. (2012). Primary non-Hodgkin's lymphoma of gingiva in a 28-year-old HIV-positive patient. *Journal of Natural Science, Biology and Medicine*.

[8] Castellarin P., Pozzato G., Tirelli G., Lenarda R. Di., Biasotto M. (2010). Oral lesions and lymphoproliferative disorders. *Journal of Oncology*.

[9] Manjunatha B. S., Gowramma R., Nagarajappa D., Tanveer A. (2011). Extranodal non-Hodgkin′s lymphoma presenting as gingival mass. *Journal of Indian Society of Periodontology*.

[10] Rozza-de-Menezes R. E., Jeronimo Ferreira S., Lenzi Capella D. (2013). Gingival anaplastic large-cell lymphoma mimicking hyperplastic benignancy as the first clinical manifestation of aids: a case report and review of the literature. *Case Reports in Dentistry*.

[11] Coiffier B. (2005). State-of-the-art therapeutics: Diffuse large B-cell lymphoma. *Journal of Clinical Oncology*.

[12] Yin H.-F., Jamlikhanova V., Okada N., Takagi M. (1999). Primary natural killer/T-cell lymphomas of the oral cavity are aggressive neoplasms. *Virchows Archiv*.

[13] Zapater E., Bagán J. V., Carbonell F., Basterra J. (2010). Malignant lymphoma of the head and neck. *Oral Diseases*.

[14] Alexander D. D., Mink P. J., Adami H.-O. (2007). The non-Hodgkin lymphomas: A review of the epidemiologic literature. *International Journal of Cancer*.

[15] Inchingolo F., Tatullo M., Abenavoli F. M. (2011). Non-Hodgkin lymphoma affecting the tongue: Unusual intra-oral location. *Head & Neck Oncology*.

[16] Jayakrishnan R., Thomas G., Kumar A., Nair R. (2008). Non-Hodgkin's lymphoma of the hard palate. *Journal of Oral and Maxillofacial Pathology*.

[17] Marrelli M., Tatullo M., Dipalma G., Inchingolo F. (2012). Oral infection by staphylococcus aureus in patients affected by white sponge nevus: A description of two cases occurred in the same family. *International Journal of Medical Sciences*.

[18] Bhattacharyya I., Chehal H. K., Cohen D. M., Al-Quran S. Z. (2010). Primary diffuse large B-Cell lymphoma of the oral Cavity: Germinal center classification. *Head & Neck Pathology*.

[19] Tatullo M., Marrelli M., Amantea M. (2015). Bioimpedance detection of Oral Lichen Planus used as preneoplastic model. *Journal of Cancer*.

